# Long-term conservative management of a mid-root fracture in an immature maxillary central incisor: a 12-year follow-up case report

**DOI:** 10.3389/fped.2026.1962051

**Published:** 2026-10-21

**Authors:** Ling Xie, Yongjie Wei

**Affiliations:** 1 Department of Pediatric Dentistry, Hefei Stomatological Hospital, Hefei, China; 2 Anhui Medical University, Hefei, China

**Keywords:** immature permanent incisor, long-term follow-up, mid-root fracture, pulp sensibility testing, splinting

## Abstract

**Background:**

Traumatic injuries to immature permanent teeth present a clinical challenge because treatment decisions may influence continued root development, pulp health, and long-term tooth survival. Conservative management aimed at preserving pulpal and periodontal tissues is an important consideration when clinical and radiographic findings indicate continued biological healing.

**Case presentation:**

A 7-year-old boy presented with traumatic displacement and a mid-root fracture of the immature maxillary right central incisor (#11) following a fall. The tooth was repositioned and stabilized with a flexible wire–composite splint. During follow-up, the tooth sustained reinjury; however, conservative management was continued because the tooth remained clinically asymptomatic and continued to respond positively to pulp sensibility testing, with no evidence of pulpal or periapical pathology. Serial clinical and radiographic examinations over 12 years demonstrated continued root development, progressive calcified healing at the fracture site, and maintenance of periodontal and tooth function.

**Outcome:**

At the 12-year follow-up, tooth #11 was asymptomatic, maintained normal crown color and function, and demonstrated a positive response to pulp sensibility testing. Radiographic examination showed complete root development with apical closure, calcified healing at the fracture site, and a normal-appearing periodontal ligament space. No evidence of pulp necrosis, inflammatory or replacement root resorption, or periapical pathology was observed.

**Conclusion:**

This case demonstrates the potential for continued root development and favorable long-term healing following conservative management of a root-fractured immature permanent tooth. The findings support careful preservation of pulpal and periodontal tissues when clinical and radiographic findings indicate ongoing biological healing. Long-term follow-up is essential to detect late complications and to guide treatment decisions while avoiding unnecessary intervention.

## Introduction

Traumatic injuries are common health problems in children and adolescents and may have long-term consequences for physical function, development, and quality of life (1). Injuries involving the maxillary anterior region are particularly important because they may affect not only oral function and aesthetics but also psychological well-being during critical stages of growth. Management of traumatic dental injuries in children therefore requires careful consideration of both immediate stabilization and long-term biological outcomes.

Immature permanent teeth represent a unique clinical condition because their development is not yet complete at the time of injury. Preservation of the health and biological function of the pulp and supporting tissues of the affected tooth is essential to allow continued root maturation and maintain long-term oral health. Unlike mature teeth, immature permanent teeth possess greater biological potential, and conservative approaches aimed at maintaining tissue integrity may avoid unnecessary invasive procedures and support natural development. Current principles in pediatric dental trauma management emphasize timely intervention, stabilization when required, and continuous monitoring of healing responses rather than immediate irreversible treatment.

Root fractures in immature permanent teeth are relatively uncommon but present significant challenges because successful outcomes depend on multiple factors (2), including fracture characteristics, tooth development stage, displacement of the injured segment, and preservation of pulp health. Although current guidelines recommend repositioning and flexible stabilization as appropriate management strategies, evidence regarding the long-term outcomes of conservative treatment in immature teeth remains limited (3), particularly in cases complicated by prolonged stabilization, poor treatment compliance, or subsequent traumatic events. Long-term clinical observations are valuable for understanding the healing capacity of developing permanent teeth and for guiding decision-making in pediatric trauma care.

Here, we report a 12-year follow-up case of an immature permanent maxillary central incisor with a mid-root fracture in a child who was successfully managed with a conservative approach. Despite initial displacement, prolonged splinting, and subsequent reinjury, the affected tooth continued to respond positively to pulp sensibility testing, achieved complete root development with apical closure, and demonstrated satisfactory functional and esthetic outcomes. This case highlights the importance of preserving pulpal and periodontal tissues and adopting long-term monitoring strategies in children with traumatic injuries involving developing permanent teeth.

## Case presentation

### Initial presentation and diagnosis

On November 29, 2012, a 7-year-10-month-old boy was referred to our clinic approximately 5 h after sustaining trauma to the maxillary anterior region due to a fall at school. The patient complained of pain and increased mobility of the upper anterior teeth during mastication. His medical history and family history were unremarkable.

Extraoral examination revealed a lower lip laceration accompanied by mild swelling. Intraoral examination showed partial extrusion of the maxillary right central incisor (#11) with grade 2 (moderate) mobility; both central incisors exhibited tenderness to percussion and sulcular bleeding. The adjacent maxillary left central incisor (#21) exhibited increased mobility without displacement.

Initial periapical radiographic examination demonstrated an oblique mid-root fracture involving the right maxillary central incisor (#11). The fracture line extended obliquely through the middle third of the root, with labio-palatal displacement of the coronal fragment and widening of the periodontal ligament space (Figure 1). Root development was incomplete, with an immature open apex. The adjacent tooth (#21) showed no evidence of fracture and was diagnosed with concussion injury.

**Figure 1:**
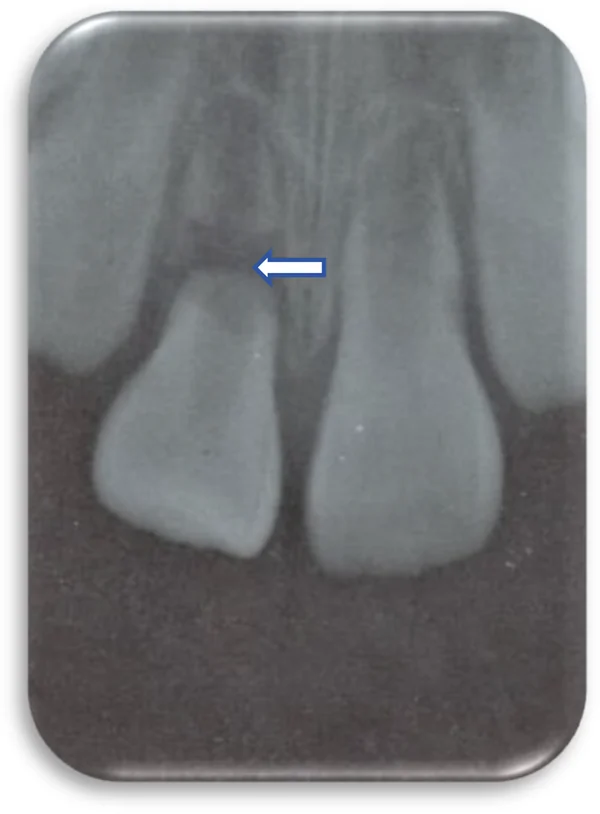
Preoperative periapical radiograph obtained on November 29, 2012, showing an oblique mid-root fracture of tooth #11 (arrow). The fracture line extends from the mesial aspect coronally toward the distal aspect apically, with displacement of the coronal fragment and widening of the periodontal ligament (PDL) space.

Considering the patient’s young age, incomplete root development, and the potential for continued biological maturation, conservative management aimed at preserving the pulpal and periodontal tissues and maintaining the natural developmental potential of the affected tooth was selected.

### Initial treatment and stabilization

After gentle cleaning and irrigation of the injured area, the displaced coronal fragment of tooth #11 was carefully repositioned to achieve anatomical alignment. A passive flexible splint consisting of six braided 0.2-mm stainless steel wire strands bonded with composite resin was applied to stabilize the injured tooth while allowing physiological mobility during the healing process. The splint extended to tooth #21 and the bilateral primary canines and primary molars to enhance stability and distribute occlusal forces.

The clinical management sequence is illustrated in Figure 2. The displaced coronal fragment was first repositioned (Figures 2A,B), followed by placement of a flexible splint using wire and composite resin fixation (Figures 2C,D). Postoperative periapical radiography confirmed satisfactory repositioning and alignment of the fracture fragments (Figure 3A).

**Figure 2:**
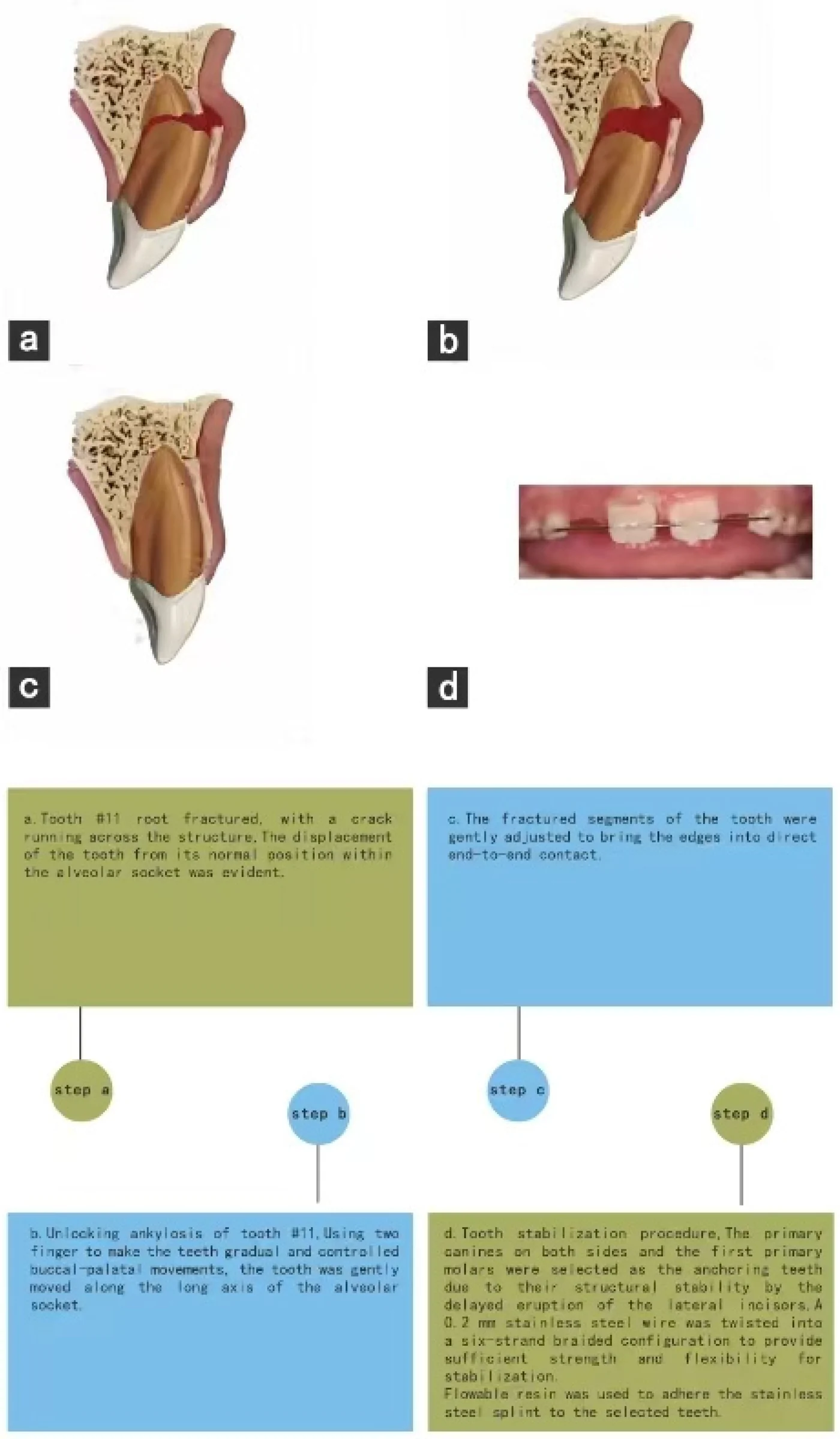
Schematic illustration of the clinical management of the mid-root fracture in tooth #11: **(a)** initial displacement of the coronal fragment; **(b)** repositioning of the coronal fragment; **(c)** application of a flexible wire–composite splint; **(d)** stabilization with adjacent teeth to allow physiological mobility.

**Figure 3:**
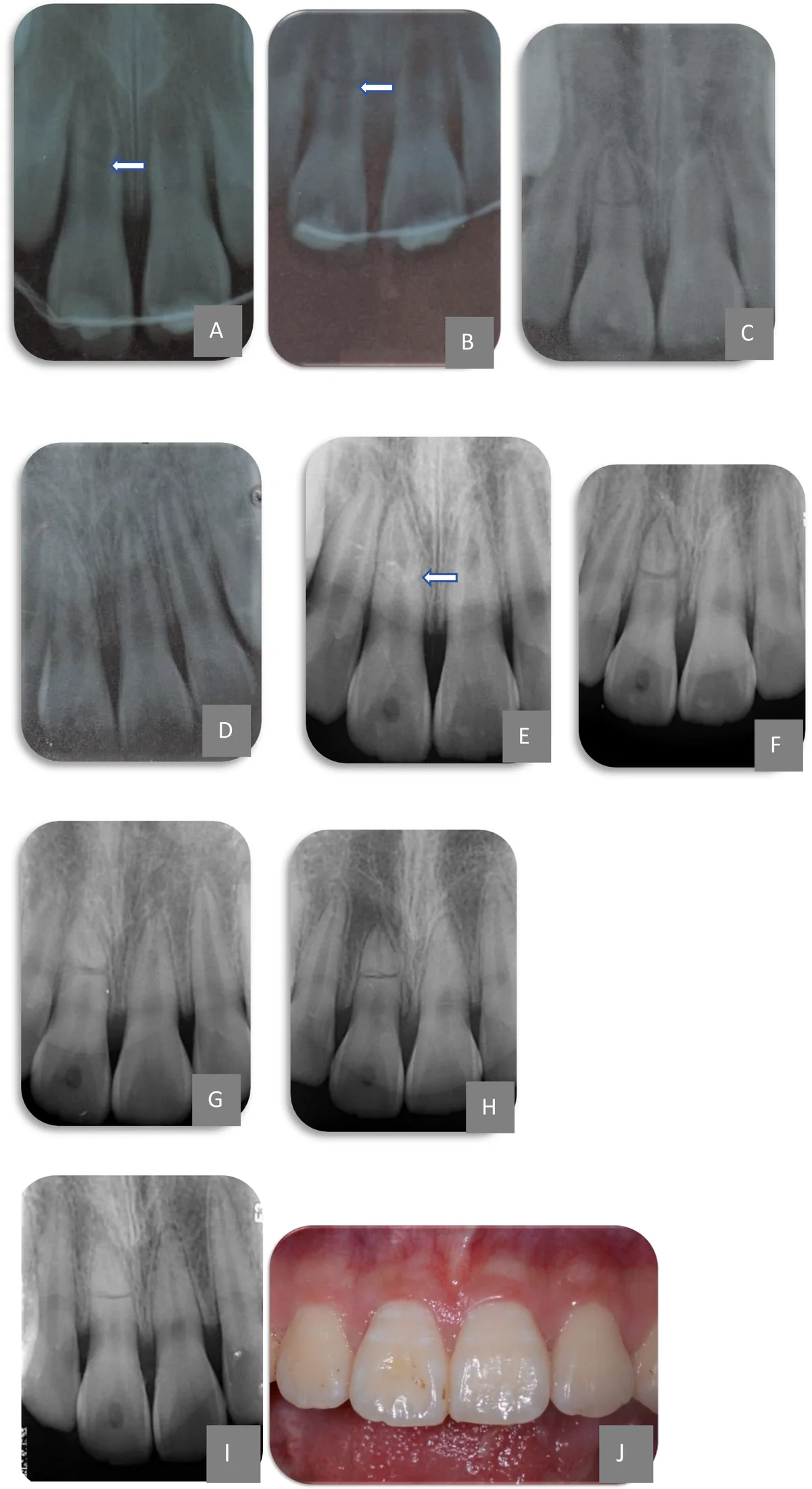
Radiographic and clinical follow-up of the fractured immature permanent incisor over a 12-year period. **(A)** Periapical radiograph obtained on December 20, 2012, immediately after splint placement, showing satisfactory repositioning and alignment of the fractured root segments. **(B)** Radiograph obtained approximately 4.5 months after trauma (April 16, 2013), demonstrating early healing changes at the fracture site with partial re-establishment of the periodontal ligament space. The fracture line remained visible. **(C)** Radiograph obtained at 9 months (August 20, 2013), showing progressive healing at the fracture site and continued root development. **(D)** Radiograph obtained at 1-year follow-up (July 16, 2014), demonstrating further root maturation and increased radiopacity at the fracture site, suggestive of hard tissue formation. **(E)** Radiograph obtained after a second traumatic episode (January 9, 2015), showing slight widening of the periodontal ligament space without displacement of fracture fragments or signs of periapical pathology. **(F)** Radiograph obtained on August 21, 2015, demonstrating continued hard tissue healing and ongoing root maturation. **(G)** Radiograph obtained on June 30, 2016, showing progressive healing with decreased visibility of the fracture line. **(H)** Radiograph obtained on August 29, 2019, demonstrating stable periodontal conditions and continued root maturation. **(I)** Periapical radiograph obtained at the 12-year follow-up (February 11, 2025), showing complete root development with apical closure, a maintained periodontal ligament space, and long-term stability of the fractured tooth. **(J)** Intraoral photograph obtained at the same visit, demonstrating satisfactory esthetic appearance of tooth #11, normal crown coloration, and healthy surrounding gingival tissues.

The patient and his parents received instructions regarding avoidance of anterior tooth loading, maintenance of oral hygiene, and the importance of regular clinical and radiographic follow-up.

### Early healing phase

During the early follow-up period, clinical management was complicated by limited patient compliance and repeated splint breakage; the splint was repaired or replaced as needed, although the dates of individual breakage episodes were not consistently recorded in the clinical records. The splint, placed on the day of injury (November 29, 2012), therefore remained in place for approximately 5 months and was removed on April 16, 2013—considerably longer than the approximately 4 weeks generally recommended for mid-root fractures (3). The prolonged stabilization resulted from the breakage episodes and delayed attendance rather than from a deliberate protocol.

At the splint-removal visit approximately 4.5 months after injury (April 16, 2013), the mobility of tooth #11 had decreased. Although the patient reported occasional spontaneous discomfort, pulp sensibility testing remained positive (electric pulp testing value: 15). Radiographic examination demonstrated partial re-establishment of periodontal ligament continuity, while the fracture line remained visible (Figure 3B).

Because the tooth continued to respond positively to pulp sensibility testing and no clinical or radiographic evidence of pulp necrosis, infection, inflammatory changes, or periapical pathology was identified, endodontic intervention was deferred. The patient and his parents also declined the planned endodontic access at that visit, and continued observation was therefore maintained.

At subsequent follow-up examinations, the patient remained asymptomatic. Progressive healing of the fracture site and continued root development were observed radiographically (Figures 3C,D).

### Management after reinjury

On January 9, 2015, approximately 2 years after the initial trauma, the patient experienced a second traumatic episode involving the maxillary anterior region. The precise mechanism of the second injury was not documented in the clinical records.

Clinical examination revealed mild tenderness to percussion and grade 0–1 mobility of tooth #11. However, the tooth remained responsive to pulp sensibility testing, with no crown discoloration or sinus tract formation.

Radiographic examination after reinjury showed slight widening of the periodontal ligament space without displacement of the fracture segments, root resorption, or periapical pathological changes (Figure 3E). Considering the maintained positive pulp sensibility response and absence of pathological findings, conservative monitoring was continued.

Follow-up radiographs demonstrated progressive healing of the injured tooth, with further reduction in fracture line visibility and continued root maturation (Figure 3F).

### Long-term outcome after 12 years of follow-up

Serial radiographic evaluations demonstrated continuous biological healing throughout the follow-up period. At later examinations, the fracture site gradually became less apparent, accompanied by maturation of the root structure and normalization of periodontal ligament appearance (Figures 3G,H).

At the final 12-year follow-up examination (February 2025), when the patient was 19 years old, tooth #11 remained asymptomatic and functional. Clinical examination revealed normal crown color, physiological mobility, and no tenderness to percussion, palpation tenderness, swelling, or sinus tract formation. The patient reported normal masticatory function without discomfort. Electric pulp testing remained positive (value: 10), comparable with the contralateral tooth.

The final periapical radiograph demonstrated complete root development with apical closure, a normal-appearing periodontal ligament space, and calcified healing at the fracture site (Figure 3I). According to the classification of root-fracture healing proposed by Andreasen et al. (5), the healing pattern corresponded to healing with calcified tissue (hard-tissue healing), based on the progressive increase in radiopacity and reduced visibility of the fracture line, the absence of a persistent radiolucent line, and the maintenance of a normal periodontal ligament space on serial radiographs. Intraoral examination showed satisfactory esthetic appearance and healthy surrounding gingival tissues (Figure 3J). No evidence of pulp necrosis, inflammatory root resorption, ankylosis, or periapical pathology was observed.

The chronological clinical and radiographic outcomes during the 12-year follow-up period are summarized in Table 1. The 12-year follow-up confirmed a maintained positive pulp sensibility response and continued root development with radiographic apical closure

**Table 1:** Chronological clinical and radiographic outcomes of tooth #11 during the 12-year follow-up period.

Follow-up Date	Clinical Findings (Tooth #11)	Radiographic Findings	Treatment/Intervention	Healing Pattern Classification	Pulp Sensibility Status
2012-11-29	Partial extrusion of the coronal fragment; grade 2 mobility; tenderness to percussion	Oblique mid-root fracture with widened PDL space	Repositioning and placement of a flexible wire–composite splint (six 0.2-mm wire strands)	Initial stabilization phase	Positive sensibility response
2013-04-16	Intermittent spontaneous pain; positive EPT response (15); reduced mobility	Partial re-establishment of PDL continuity; fracture line visible	Splint removal; continued observation	Ongoing healing; calcified tissue healing suspected	Positive EPT response
2013-08-20	Asymptomatic; normal percussion response	Progressive healing of fracture line; continued root development	Continued observation	Healing with calcified tissue	Positive sensibility response
2014-07-16	No pain or abnormal mobility	Increased radiopacity at fracture site; continued root maturation	Continued follow-up	Healing with calcified tissue	Positive sensibility response
2015-01-09	Mild percussion sensitivity; grade 0–1 mobility; positive pulp sensibility response	Slight widening of PDL space; incomplete root development; no periapical pathology	Conservative monitoring after reinjury	Healing with calcified tissue	Positive sensibility response
2015-08-21	Asymptomatic	Continued root maturation and reduction in fracture line visibility	Continued observation	Healing with calcified tissue maintained after reinjury	Positive response after reinjury
2016-06-30	Normal clinical findings	Further healing of fracture site and PDL normalization	Routine follow-up	Healing with calcified tissue	Positive sensibility response
2019-08-29	Asymptomatic; normal crown color; physiological mobility	Stable root maturation; normal PDL space; fracture line barely visible	Routine follow-up	Advanced healing with calcified tissue	Positive sensibility response
2025-02-11	Positive EPT response (10); no tenderness, mobility, or discoloration	Complete root development with apical closure; intact PDL space; no root resorption or periapical pathology	Long-term follow-up	Complete healing with calcified tissue	Positive response; complete root development with apical closure

EPT, Electric pulp test. Mobility grading: 0 = physiological mobility; 1 = slight mobility; 2 = moderate mobility; 3 = severe mobility. Healing pattern classification was based on the criteria proposed by Andreasen et al.: Healing with calcified tissue. Healing with interposition of connective tissue. Healing with interposition of bone and connective tissue. Non-healing associated with pulp necrosis.

## Discussion

Traumatic injuries involving the oral and maxillofacial region are common during childhood and may have long-lasting effects on oral function, aesthetics, and quality of life. Management of traumatic injuries in children differs from that in adults because treatment decisions must consider not only immediate injury control but also ongoing growth and development. In immature permanent teeth, preserving biological function and avoiding unnecessary invasive procedures are particularly important because continued root maturation and tissue adaptation may significantly influence long-term outcomes.

The present case demonstrated successful long-term functional preservation of an immature permanent incisor following traumatic mid-root fracture. Over a 12-year follow-up period, the affected tooth maintained a positive pulp sensibility response, completed root development with apical closure, and achieved satisfactory clinical and radiographic outcomes despite several factors that could potentially compromise healing, including initial displacement of the coronal fragment, prolonged splinting, and subsequent reinjury (4, 5). These findings highlight the potential of conservative management strategies in selected pediatric trauma cases when biological healing capacity remains.

Compared with previously published reports, the principal contribution of this case lies in the duration and circumstances of the follow-up rather than in a fundamentally novel treatment approach. Long-term observations of root fractures in immature permanent teeth remain scarce: previous reports have described conservative management of root fractures in young immature permanent teeth with shorter follow-up periods (9), maintenance of pulp tissue after horizontal root fractures in maxillary incisors over 13 years (6), and a two-staged single-visit treatment of a complicated crown-root fracture with an additional horizontal mid-root fracture with a 5-year follow-up (1). In a large case series, Andreasen et al. reported 10-year survival rates of 89% for apical-third, 78% for mid-root, and 67% for cervical-third root fractures (5). The present case adds to this literature by documenting, over 12 years, continued root development to radiographic apical closure and calcified tissue healing in an immature tooth with a mid-root fracture that was additionally complicated by prolonged splinting and a second traumatic episode during the healing period.

Preservation of pulp health is a key objective in the management of traumatic injuries involving immature permanent teeth. Because pulp sensibility testing assesses neural responsiveness rather than histologic vitality, pulp status in this report was evaluated by combining sensibility testing with clinical and radiographic findings; a positive response together with the absence of signs of pulp necrosis was interpreted as evidence of preserved pulp function. Unlike fully developed teeth, immature teeth possess continued developmental potential, and maintenance of pulp function allows ongoing root maturation and strengthening of the tooth structure (6). In the present case, although transient symptoms occurred during follow-up, pulp sensibility testing remained positive and no radiographic evidence of infection, inflammatory resorption, or periapical pathology was observed. Therefore, invasive endodontic treatment was deferred, allowing the tooth to continue its natural developmental process. This approach reflects the broader pediatric principle of minimizing unnecessary intervention while preserving physiological function whenever possible.

The favorable outcome in this case may be attributed to several biological and clinical factors (7). First, timely repositioning and stabilization provided an appropriate environment for periodontal and pulpal healing. Second, the immature status of the affected tooth may have contributed to its regenerative potential and ability to continue root maturation after injury (8, 9). Serial radiographic examinations demonstrated progressive healing of the fracture site, normalization of the periodontal ligament space, and completion of root development over time. These observations emphasize that healing after traumatic injury in children is a dynamic process that may continue for many years beyond the initial treatment period.

An important feature of this case was the occurrence of a second traumatic episode during the healing period. Reinjury is generally considered a potential risk factor because additional mechanical stress may interfere with periodontal healing or compromise pulp status. However, in this patient, the second trauma resulted in only temporary clinical symptoms without loss of the positive pulp sensibility response or development of pathological changes. This suggests that continued observation may be appropriate in selected cases when clinical and radiographic findings remain favorable. Decisions regarding further intervention should be based on biological indicators rather than traumatic history alone.

Another notable aspect was the prolonged stabilization period caused by limited patient compliance and repeated splint breakage (10–14). Current guidelines recommend flexible splinting for approximately 4 weeks for mid-root fractures (3); the 5-month stabilization in this case substantially exceeded this recommendation and resulted from repeated splint breakage and limited compliance rather than from a deliberate protocol. Although prolonged splinting did not prevent favorable healing in this patient when a positive pulp sensibility response was preserved and appropriate follow-up was maintained (15, 16), this single observation cannot demonstrate the safety of prolonged splinting and should not be interpreted as support for routinely extending stabilization. Instead, it provides additional clinical insight into the variability of healing responses in developing teeth.

From a pediatric healthcare perspective, this case emphasizes the importance of long-term follow-up after childhood trauma. Successful management should not be evaluated only by short-term symptom resolution but also by preservation of function, normal development, and avoidance of unnecessary treatment throughout the child’s growth period. The 12-year follow-up in this case provides evidence that conservative approaches may achieve favorable long-term outcomes in selected children with traumatic injuries involving immature permanent teeth.

This report has several limitations. As a single case report, the findings cannot establish causal relationships or predict outcomes for all children with similar injuries. Treatment decisions in this case were individualized and may not apply to teeth with pulp necrosis, infection, severe displacement, or different fracture characteristics. Radiographic assessment was based on conventional two-dimensional periapical radiographs obtained without rigid standardization of projection geometry; assessments of root maturation, fracture healing, and periodontal ligament changes were therefore based on visual comparison of serial images, and terms such as ‘complete healing’ should be understood within this limitation. In addition, detailed periodontal probing records and the precise dates of individual splint breakage episodes were not retained in the clinical records. Advanced imaging modalities such as cone-beam computed tomography were not used because conventional radiographs provided sufficient information for clinical monitoring while minimizing radiation exposure in a young patient. Further longitudinal studies involving larger pediatric populations are required to better understand prognostic factors and optimize management strategies for traumatic injuries of immature permanent teeth.

## Conclusion

This case illustrates the potential for continued root development and favorable long-term healing following conservative management of a mid-root fracture in an immature permanent tooth. Over a 12-year follow-up period, the injured tooth maintained a positive pulp sensibility response, completed root development with apical closure, and preserved satisfactory functional and esthetic outcomes despite initial displacement, prolonged stabilization, and subsequent reinjury. As a single case, these findings cannot establish causation and do not indicate that conservative management should universally replace endodontic intervention in traumatized immature teeth.

For children with traumatic injuries involving developing permanent teeth, treatment decisions should consider not only immediate stabilization but also long-term biological development. Preservation of pulpal and periodontal tissues and avoidance of unnecessary invasive procedures may support natural maturation and functional preservation of the affected tooth when clinical and radiographic findings indicate ongoing biological healing. Careful clinical and radiographic monitoring remains essential to guide individualized management and optimize long-term outcomes.

### Learning points

Traumatic injuries involving immature permanent teeth in children require treatment strategies that consider both injury management and ongoing growth and development.

Preservation of pulp health should remain a primary goal whenever possible, as maintaining a positive pulp sensibility response together with the absence of periapical pathology may allow continued root maturation and long-term tooth survival.

Favorable healing may occur after conservative management even in complex clinical situations involving fracture displacement, prolonged stabilization, and subsequent reinjury, provided that a positive pulp sensibility response and periodontal health are maintained.

Long-term follow-up is essential in pediatric dental trauma because healing and developmental changes may continue for many years after the initial injury.

Conservative, minimally invasive approaches may represent an important strategy for preserving natural tissues and improving long-term functional outcomes in growing children.

## Patient perspective

At the time of the initial injury, the patient was a young child undergoing active dental development. Throughout the follow-up period, the patient and his family were informed about the healing process and the importance of maintaining regular clinical evaluations.

After 12 years of follow-up, the patient reported satisfaction with the preservation of his natural tooth and the avoidance of additional invasive dental procedures. The maintenance of normal tooth appearance and function during adolescence and early adulthood was considered an important outcome by both the patient and his family.

This long-term follow-up experience highlights the potential value of conservative management strategies in pediatric traumatic dental injuries, particularly when preservation of pulp sensibility, periodontal health, and natural development can be achieved.

## Data Availability

The original contributions presented in this study are included in the article. Further inquiries regarding the clinical data should be directed to the corresponding author.
